# Construction of ferulic acid modified porous starch esters for improving the antioxidant capacity

**DOI:** 10.1039/d1ra08172a

**Published:** 2022-02-02

**Authors:** Shenggui Zhang, Haiyan Li, Min Li, Guopeng Chen, Yunxiang Ma, Yue Wang, Jinfeng Chen

**Affiliations:** College of Food Science and Engineering, Gansu Agricultural University Lanzhou 730070 China; Gansu Provincial Key Laboratory of Arid Land Crop Science Lanzhou 730070 China; State Key Laboratory of Applied Organic Chemistry, College of Chemistry and Chemical Engineering, Lanzhou University Lanzhou 730000 Gansu China; College of Science, Gansu Agricultural University Lanzhou 730070 China

## Abstract

For exploration of a type of synthetic antioxidant dietary fiber (ADF), porous starch (PS), modified by esterification with ferulic acid (FA) moieties, was synthesized successfully, with different degree of substitution (DS). The ester linkage of FA modified PS was confirmed by ^13^C solid-state NMR and FT-IR. XRD analysis showed that starch ferulates had a different crystal structure from the V-type pattern of native starch, suggesting that the starch gelled during the esterification reaction, then re-crystallized into a different structure. Morphological studies revealed that FA modified PS esters had a different morphology of irregular beehive-like and dense fibrous-like structures compared with that of native starch. *In vitro* antioxidant assays showed that starch ferulates had excellent antioxidant capacity. In particular, FA modified PS esters had a much higher antioxidant capacity than free FA in the β-carotene–linoleic acid assay. This study has advanced the technology of using porous starches for modifying the biological activity of an antioxidant polyphenol. We expect this work would inspire further studies on the interactions of phenolics with other food ingredients in the food industry.

## Abstract 2

For exploration of a type of synthetic antioxidant dietary fiber (ADF), porous starch (PS), modified by esterification with ferulic acid (FA) moieties, was synthesized successfully, with different degree of substitution (DS).

## Graphical abstract

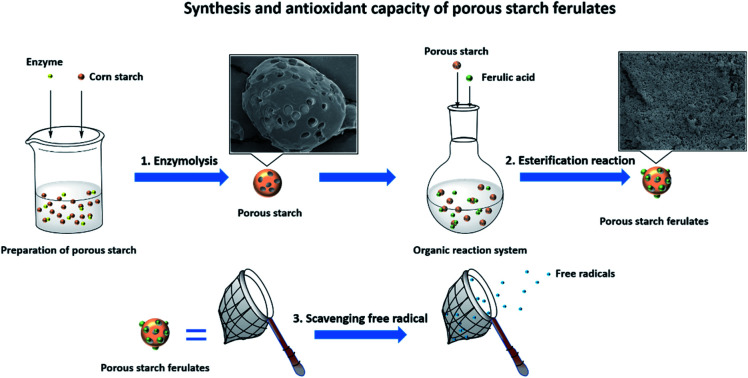

## 1. Introduction

Epidemiological studies indicate that regular consumption of fruits and vegetables is associated with lower risk of diseases including stroke, cancer and other chronic diseases.^1^ Naturally occurring antioxidant dietary fibers (ADFs), in which antioxidant polyphenolic compounds are linked to dietary-fiber polysaccharides, combine the beneficial effects of both dietary fiber and natural antioxidants,^2^ and appear particularly effective for the prevention of chronic and degenerative diseases.^3^ ADFs are an abundant source of phenolic antioxidants such as phenolic acids (ferulic acid, caffeic acid, *etc.*), flavonoids (hesperidin, catechins, *etc.*) and tannins.^2,4,5^ Recent research has focused on synthetic ADFs and antioxidant polysaccharide derivatives, which combine polyphenols, such as ferulic acid, with starch,^6^ alginates^7^ and chitosan.^8^

Ferulic acid (FA), an antioxidant phenolic acid found in many plants, is one of the most widely occurring of the hydroxycinnamic acids.^9^ The antioxidant activity, free radical-scavenging capacities and anti-inflammatory activity of ferulic acid are well-established. However, free ferulic acid from oral ingestion does not enter the intestinal circulation and cannot easily reach the colon after oral ingestion. Besides, intravenous administration has low bioavailability to the circulatory system and consequently, little *in vivo* biological activity.^10^ Starch-based carriers, which are almost completely degraded in the colon, are an effective approach for improving the bioavailability of ferulic acid in ADFs; the colon microflora releases ferulic acid from starch ferulates much more extensively than from wheat bran.^6^ Starch ferulates, as a food-ingredient, have better functional and physicochemical properties than native starch, including lower viscosity, higher water holding capacity and a reduced tendency for retrogradation during low temperature storage.^11,12^ Starch ferulates, therefore, showed a feasible alternative strategy to improve starch physicochemical properties with higher bioavailability of ferulic acid.^6,10^

Porous starch (PS), prepared by physical, chemical, or enzyme treatment, is a modified starch that contains micron-sized pores extending throughout the granules.^13^ The porous structure of PS gives it a much larger specific surface area and many more chemically reactive sites than native starch, consequently chemical-modification of PS results in a much higher degree of substitution (DS).^14^ PS is therefore, a very suitable substrate for chemical modification with FA. To the best of our knowledge, there are few reports on the detailed chemical and morphological characterization of PS modified with FA.

In this study, porous starch modified with FA (donated as FA@PS) was synthesized successfully, using *N*,*N*′-carbonyldiimidazole (CDI) to promote the esterification reaction of starch hydroxyl groups with the carboxyl group of ferulic acid. The ester linkage of FA@PS was confirmed by ^13^C solid-state NMR and Fourier transform infrared spectroscopy (FT-IR). The crystalline structure and morphology were characterized by X-ray diffraction analysis (XRD) and scanning electron microscopy (SEM), respectively. The thermal properties of FA@PS were also determined. The antioxidant capacity of FA@PS was characterized by four *in vitro* antioxidant assays, including assays of scavenging capacity for 2,2-diphenyl-1-picrylhydrazyl (DPPH), ferric reducing antioxidant power (FRAP), reducing power and the β-carotene–linoleic acid assay. The findings from this study will provide improved understanding of ADFs to facilitate new applications for lowering the risk of chronic disease and expanding the applications of functional polysaccharides.

## 2. Materials and methods

### 2.1. Materials

Corn starch, α-amylase (AM, 50 U mg^−1^), amyloglucosidase (AMG, 100 U mL^−1^), were purchased from Shanghai Yuanye Biotechnology Co., Ltd (Shanghai, China). Ferulic acid (99%, HPLC grade), *N*,*N*′-carbonyldiimidazole (CDI), were purchased from Macklin Biochemical Technology Co. Ltd (Shanghai, China). All the reagents used were of analytical grade unless otherwise stated.

### 2.2. Preparation of porous starch (PS)

PS was prepared as described previously^13^ with a minor modification. Native corn starch (10.0 g) was suspended in a mixture of phosphate buffer (60 mL, pH 6.6) and acetate buffer (20 mL, pH 4.5), by stirring in a water bath at 40 °C for 20 min. The enzymes, α-amylase and amyloglucosidase (mass ratio 6 : 1), were added into the suspension the mixture was shaked in a water bath at 50 °C for 24 h, then the pH was adjusted to 10 with 1 M NaOH solution. The suspension was centrifuged (7000 rpm, 15 min, at 4 °C), washed with distilled water, then dried at 50 °C.

### 2.3. Preparation of starch ferulates

Starch ferulates were synthesized as described previously.^15^ Imidazolide-activated FA was prepared by FA and CDI (mole ratio 1 : 1) in anhydrous dimethylsulfoxide (DMSO) (20 mL), with magnetic stirring for 20 min, under nitrogen. The mixture was then maintained at 60 °C with continuous stirring (300 rpm) for 16 h to complete the reaction. Native starch (NS), or PS (1.0 g) was then added directly to the imidazolide-activated FA solution. The mixture was then maintained at 90 °C with continuous stirring (300 rpm) for 7 h, then isopropyl alcohol (40 mL) was added to precipitate the product. The reaction product was recovered by centrifugation at 4000 rpm for 20 min, then washed twice with isopropanol to remove unreacted FA, CDI, and imidazolide-activated FA. The product was dissolved in DMSO, then dialyzed against DMSO for 24 h, using molecular weight cut-off 8000–14 000 Da dialysis tubing. The product was dissolved in distilled water, then dialyzed against distilled water for 48 h to remove residual DMSO and isopropanol. Finally, a yellowish, powdery final product was obtained by lyophilization. Starch ferulates with different degrees of substitution were designated as 0.11FA@NS, 0.21FA@PS, 0.37FA@PS and 0.43FA@PS, respectively (the numbers represent DS).

### 2.4. Determination of the degree of substitution (DS)

The DS of starch ferulates was determined as described previously, with minor modifications.^16^ Sample (∼0.1 g) was accurately weighed, then dissolved in NaOH (2 mol L^−1^, 20 mL) with a magnetic stirring for 2 h, to fully hydrolyze the ester linkages. HCI solution (6 mol L^−1^) was added dropwise to adjust the pH to 1.6, then the hydrolysate was exhaustively extracted with ethyl acetate, and the organic layer collected and rotary-evaporated to dryness. The dried extract was dissolved in methanol (100 mL) and FA quantified by HPLC analysis, by absorption at 322 nm, and elution with methanol and 1% (v/v) aqueous acetic acid in a volume ratio of 45 : 55 at a flow rate of 1.0 mL min^−1^. DS was calculated according to the equation below:
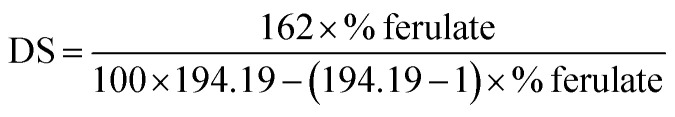
where, 162 refer to the molecular weight of anhydroglucose unit; 194.19 refer to molecular weight of ferulate group; % ferulate refer to percentage of ferulate in samples.

Reaction efficiency referred to the ratio of DS measured by HPLC, to theoretical DS (molar ratio of starch ferulate to anhydroglucose residue).

### 2.5. NMR spectroscopy

Solid-state NMR measurements were carried out on a Bruker WB Avance II 400 MHz spectrometer. The ^13^C CP/MAS NMR spectra were recorded with a 4 mm double-resonance MAS probe and with a sample spinning rate of 10.0 kHz. The ^1^H NMR spectra were recorded using a Bruker D8 ADVANCE Spectrometer (Bruker, Germany) at 400 MHz. The samples were dissolved in deuterated dimethyl sulfoxide (DMSO-d_6_) at 50 °C.

### 2.6. Fourier transform infrared (FT-IR) spectroscopy

The infrared spectra of NS, PS, FA@NS and FA@PS were determined by FT-IR spectroscopy (Nicolet NEXUS 670, Thermo Company, Waltham, USA). The KBr and samples were mixed and ground in an agate mortar until no obvious graininess was observed. Scanning was performed from 4000–450 cm^−1^, scanning mode of 32 times per s.

### 2.7. XRD analyse

The crystal structures of NS, PS, FA@NS and FA@PS were characterized by X-ray diffract meter (XRD-6000, Shimadzu, Japan). The test conditions were as follows: voltage: 40 kV; current: 40 mA; scanning range: 5–35°; scanning speed: 2° min^−1^; scanning step size: 0.06°; scanning method: continuous.

### 2.8. SEM image analysis

The morphology of NS, PS, FA@NS and FA@PS were observed using SEM instrument (JSM-6701F, Electron Optics Inc., Tokyo, Japan). Samples powder were stuck on a specimen stage, then coated with gold in a vacuum chamber. The shape and surface characteristics of the samples were observed at an accelerating voltage of 5 kV.

### 2.9. Thermal properties

Thermal properties were measured using a DSC instrument (DISCOVERY 25, TA Instruments, New Castle, DE) and thermogravimetric analysis (DISCOVERY 55, TA Instruments, USA).

### 2.10. Evaluation of antioxidant capacity

DPPH scavenging capacity was determined as described previously, with minor modifications.^17^ FRAP capacity was determined as described previously.^18^ Reducing power was determined as described previously.^19^ Inhibition of linoleic acid peroxidation was determined by a β-carotene bleaching test, with some modifications.^20^

### 2.11. Statistical analysis

The whole experiments were assayed in triplicate and results were expressed as the mean and the standard deviation. The one-way analysis of variance (ANOVA) test was analyzed using the SPSS 22.0 package, following Duncan's multiple-range test. Significance was defined at *p* < 0.05.

## 3. Results and discussion

### 3.1. DS of starch ferulates

The FA@NS and FA@PS possessing different DS were synthesized according to the methods above, as summarized in Table 1. The reaction efficiencies of starch ferulates with DS values of 0.11, 0.21, 0.37 and 0.43 were 19.49%, 24.25%, 31.24% and 36.18%, respectively. The DS was enhanced as the increasing molar number of FA, which could be interpreted that the availability of the activated FA in the vicinity of the starch molecules.^12^ FA@PS has a larger DS (DS = 0.43) than that of FA@NS, which was derived from additional pores and channels within the PS structure that provided a larger specific surface area and more reactive sites with FA.

**Table 1:** DS of starch ferulates

Samples	*M*_FA_ a	*M*_starch_ a	DS	Reaction efficiency (%)
0.11FA@NS	0.0038	0.00617	0.11	19.49
0.21FA@PS	0.0038	0.00617	0.21	24.25
0.37FA@PS	0.0061	0.00617	0.37	31.24
0.43FA@PS	0.0123	0.00617	0.43	36.18

a *M*_FA_ and *M*_starch_ presented the molar number of ferulic acid and starch applied.

### 3.2. NMR

#### 3.2.1 ^1^H NMR spectra

**Fig. 1:**
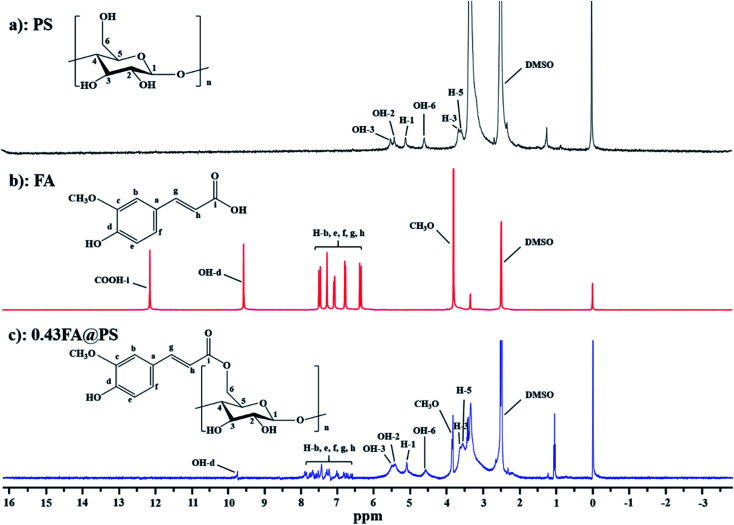
^1^H NMR spectra of PS (a), FA (b) and porous starch ferulate with DS of 0.43 (c). These peaks at 6.54–8.06 ppm were attributed to the methine protons of feruloyl, indicating that porous starch ferulate was obtained successfully.

To confirm the formation of starch ferulate, ^1^H NMR spectra were recorded, of PS, FA and FA@PS with DS of 0.43 (Fig. 1). The protons of the glucosyl residues of PS appeared at 5.11 ppm (H-1), 5.42 ppm (OH-2), 5.52 ppm (OH-3), 4.59 ppm (OH-6) and 3.15–3.65 ppm (H-2, H-3, H-4, and H-5) (Fig. 1a).^21^ The aromatic and C=C protons of FA appeared between 6.54 and 8.06 ppm (Fig. 1b).^12^ Compared with the spectrum of PS, FA@PS had additional signals, including multiple signals between 6.54 and 8.06 ppm (methine protons of the feruloyl moiety), a strong peak at 3.85 ppm (-OCH_3_), and a weak peak at 9.76 ppm (phenolic –OH) (Fig. 1c). The presence of these signals confirmed the successful synthesis of FA@PS. In addition, the absence of a FA carboxylic proton signal (∼12.14 ppm) in FA@PS, both confirmed the formation of an ester linkage and the complete removal of unreacted FA.

#### 3.2.2 ^13^C solid-state NMR spectra

**Fig. 2:**
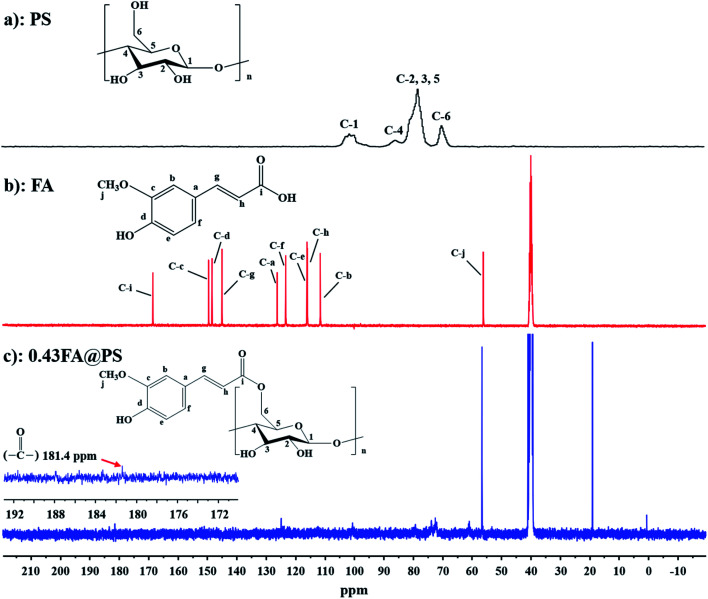
Solid-state ^13^C CP/MAS NMR spectra of PS (a), FA (b) and porous starch ferulate with DS of 0.43 (c). The peak at 181.4 ppm was attributed to the carbonyl carbon, indicating that porous starch ferulate was obtained successfully.

To further confirm the formation of an ester linkage, solid-state ^13^C CP/MAS NMR spectra were recorded of PS, FA and FA@PS (DS = 0.43) (Fig. 2). In the high field, the peaks centered at 62.0 ppm, 72.4–82.6 ppm and 101.7 ppm were attributed to signals of C-6, C-2, 3, 4, 5 and C-1, respectively, of the starch glucosyl residue (Fig. 2a).^22,23^ The signals for the FA carbon atoms were assigned as reported previously (Fig. 2b).^24,25^ The peak corresponding to the ester group carbonyl carbon was observed at 181.4 ppm in the down field,^26^ considerably shifted from that of the FA carboxyl carbonyl (168.5 ppm), further confirming ester formation between FA and starch hydroxyl groups.

### 3.3. Fourier transform infrared (FT-IR) spectroscopy

**Fig. 3:**
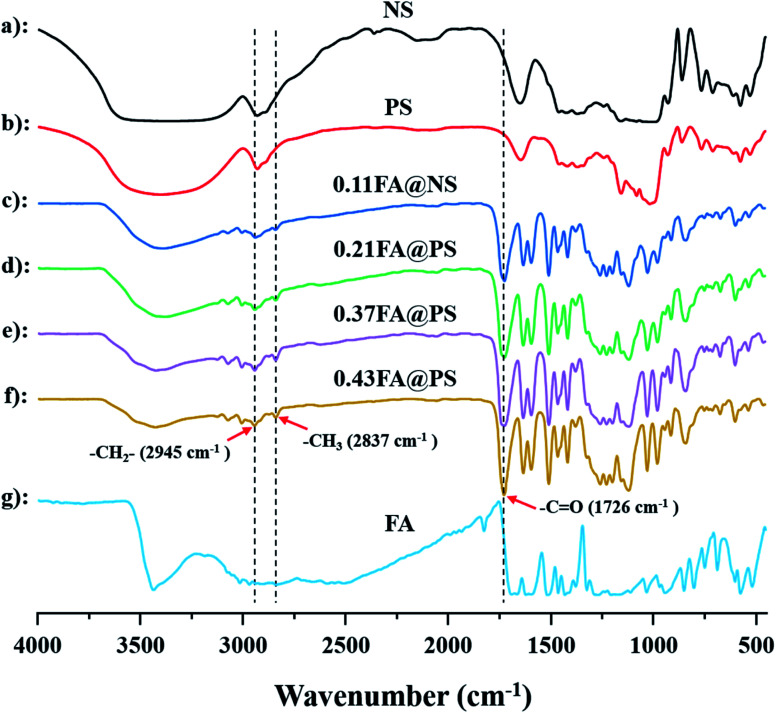
FT-IR spectra of NS, PS, FA and starch ferulate with different DS (a: NS; b: 0.11FA@NS; c: 0.21FA@PS; d: 0.37FA@PS; e: 0.43FA@PS; f: FA); the bands of starch ferulate at 1726 cm^−1^ was attributed to the stretching vibration of C=O, indicating the successful formation of the ester linkage.

Meanwhile, the ester formation was also confirmed in starch ferulate by FT-IR spectroscopy (Fig. 3). The spectrum of NS was almost identical to that of PS, indicating that the enzymatic hydrolysis had little, or no effect on the starch structure, apart from creating pores.^27^ Compared with the spectra of non-esterified NS and PS, a new absorption band at 1726 cm^−1^, corresponding to the C=O of the feruloyl ester, was observed in those of the starch ferulates (Fig. 3c–f).^10^ In addition, two bands at 2933 cm^−1^ and 2849 cm^−1^ were attributed to methyl and methylene C–H stretching, and those around 1450–1600 cm^−1^ to skeletal vibrations of the aromatic ring, of the feruloyl substituents.^28^ Comparing the spectra of FA@NS and FA@PS, the C=O adsorption band (1726 cm^−1^) of FA@PS was observed with the higher intensity than that of FA@NS, suggesting that enzymatic hydrolysis increased the extent of esterification, resulting from the larger surface area of PS. These results further confirmed that FA was successfully introduced into both NS and PS by the esterification reaction.

### 3.4. X-ray diffraction studies (XRD)

**Fig. 4:**
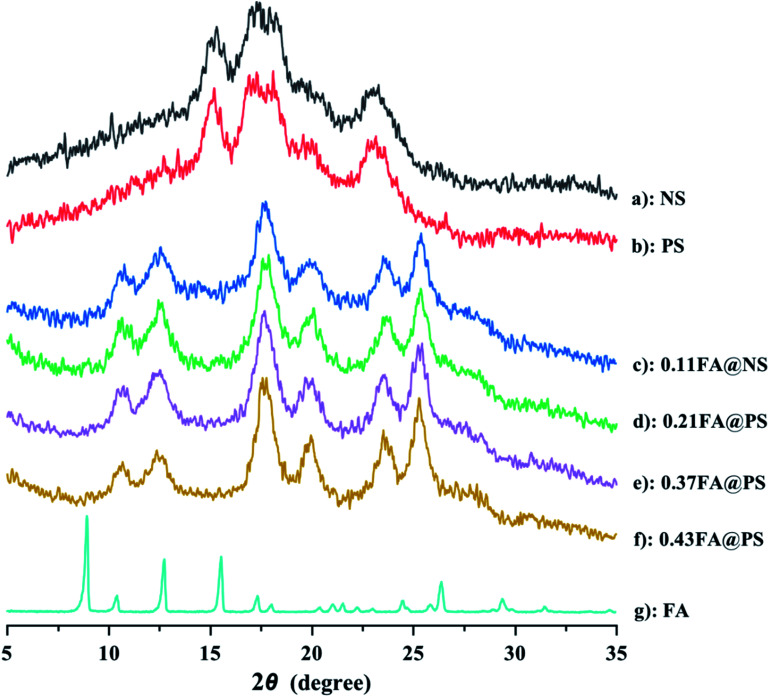
XRD patterns of NS, PS, starch ferulate with different DS and FA (a: NS; b: PS; c: 0.11FA@NS; d: 0.21FA@PS; e: 0.37FA@PS; f: 0.43FA@PS; g: FA). The result indicated that the crystalline structure was fully changed after esterification.

To compare the crystal structure of PS before and after esterification reaction with FA, XRD analysis was carried out on NS, PS, FA and the starch ferulates (Fig. 4). The NS had a typical A-type crystal structure (Fig. 4a), showing strong diffraction peaks at 15.1°, 17.0°, 18.0° and 23.1°. The XRD pattern of PS was almost identical to that of NS (Fig. 4b), indicating that the crystal structure was unchanged after enzyme treatment.^29^ FA (Fig. 4g) had sharp diffraction peaks at 9.0°, 10.4°, 12.8°, 15.6°, 17.4°, 18.1°, 25.0° and 26.5°. The XRD patterns of the starch ferulates (Fig. 4c–f) were very similar, with diffraction peaks at 12.5°, 20.1°, 17.8°, 23.0° and 25.3°, indicating that a major rearrangement of starch chains occurs after esterification. The feruloyl groups esterified with some of the hydroxyl groups on the starch chains, disrupting some of the intermolecular hydrogen bonds, thereby changing the crystalline structure.^22^ The new structure induced by the esterification, was not the same as the reported V-type pattern of NS.^21,30^ The new diffraction peaks appear to be related to the decrease in water solubility of the product and the formation of aggregates with an ordered structure, like “shell–core” polymeric micelles, with a diffraction pattern different from that of V-type retrograded starch.^31^

### 3.5 Scanning electron microscopy (SEM)

**Fig. 5:**
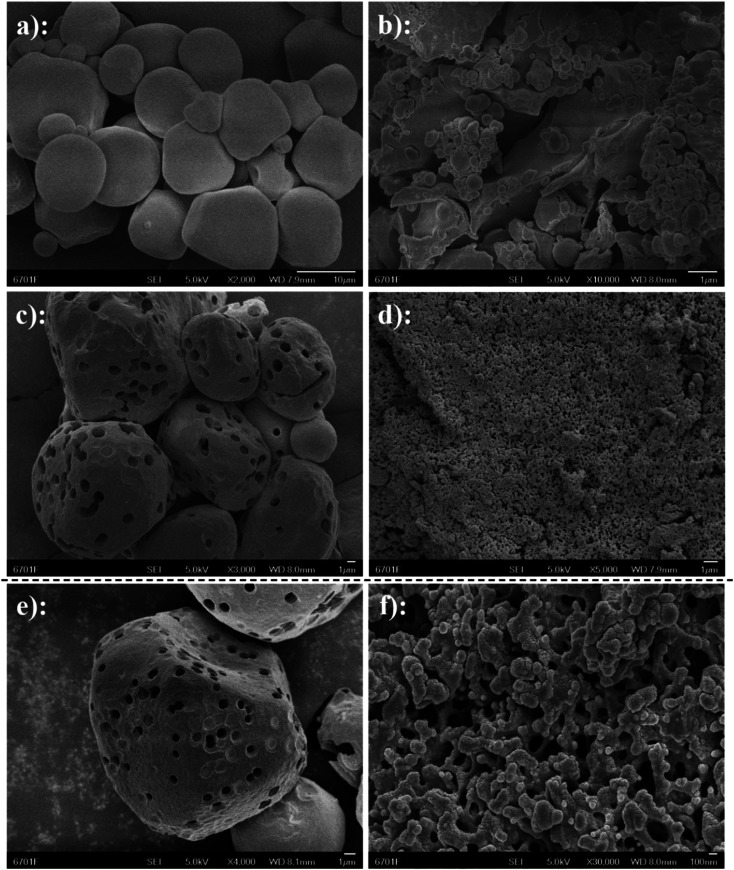
SEM images of NS (a, ×2000) and FA@NS (b, ×10 000), images of PS (c, ×3000) and FA@PS (d, ×5000), images of PS with magnification of 4000 times (e) and FA@PS with magnification of 30 000 times (f); the SEM image of FA@PS after esterification showed dense small pores obviously, indicating that smaller pore structure was formed by porous structure after post-treatment.

To observe the morphology of starch ferulate, the microstructure was analyzed using SEM (Fig. 5). NS (Fig. 5a) had irregular, rounded granules, with a relatively smooth surface and a narrow size-range. NS granules after esterification (Fig. 5b), had a much wider size-range and a highly-irregular morphology, apparently resulting from gelatinization and aggregate formation, during the esterification treatment.^10^ PS (Fig. 5c) exhibited relatively few, but large pores, some shallow, but others extending into the interior of the starch granules. These are responsible for the larger specific surface area,^32^ which makes PS more suitable for surface chemical modification than NS. At higher magnification (Fig. 5e), different concentric layers can be distinguished within the pores. FA@PS (Fig. 5d) completely lost the granular structure of PS, exhibiting large, microporous aggregates. At high magnification (Fig. 5f), the surface of FA@PS appears porous with a very large specific surface area, compared with FA@NS. This indicates that the esterification reaction occurs not only on the surface of the starch, but also inside the starch granules, disrupting their internal structure through gelatinization,^33^ which is consistent with the XRD results.

### 3.6. Thermal properties

#### 3.6.1 Differential scanning calorimetry (DSC) analysis

The thermal properties of NS, PS and starch ferulates were determined by differential scanning calorimetry (DSC) to detect possible changes in the physical state of the starch structure (Table 2). Compared with NS, the values of *T*_o_, *T*_p_ and Δ*H* of PS were slightly higher, which suggested that PS had a slightly higher degree of crystallinity,^34^ indicating that the structure of PS was essentially the same as NS. Dense packing hinders both heat and mass transfer, which results in higher melting temperatures. *T*_o_, *T*_p_ and *T*_c_ markedly increased for all the starch ferulates, but Δ*H* decreased. This may result from disruption of double-helical structures in the amorphous regions of the starch granules during esterification,^35^ resulting in a different crystal structure in the starch ferulates, consistent with the XRD results above.

**Table 2:** Thermal properties of NS, PS and starch ferulates^a^

Samples	*T*_o_ (°C)	*T*_p_ (°C)	*T*_c_ (°C)	Δ*H* (°C)
NS	66.16 ± 0.0214^d^	71.58 ± 0.0157^d^	77.08 ± 0.2601^d^	9.25 ± 0.0383^b^
0.11FA@NS	125.39 ± 0.0356^c^	130.5 ± 0.0242^c^	140.58 ± 0.1660^b^	6.59 ± 0.1370^d^
PS	67.98 ± 0.0132^d^	72.13 ± 0.0146^d^	77.05 ± 0.0214^d^	10.81 ± 0.0269^a^
0.21FA@PS	128.00 ± 0.0124^c^	128.91 ± 0.0223^c^	134.87 ± 0.1253^c^	6.29 ± 0.1668^d^
0.37FA@PS	132.11 ± 0.0221^b^	133.35 ± 0.0164^b^	138.47 ± 0.0201^b^	7.21 ± 0.0206^c^
0.43FA@PS	136.70 ± 0.0216^a^	137.81 ± 0.0203^a^	143.63 ± 0.0141^a^	8.26 ± 0.0232^c^

a Values are given as mean ± standard deviation. Different superscript letters in the columns indicate significant difference (*p* < 0.05).

#### 3.6.2 Thermogravimetric analyses

**Fig. 6:**
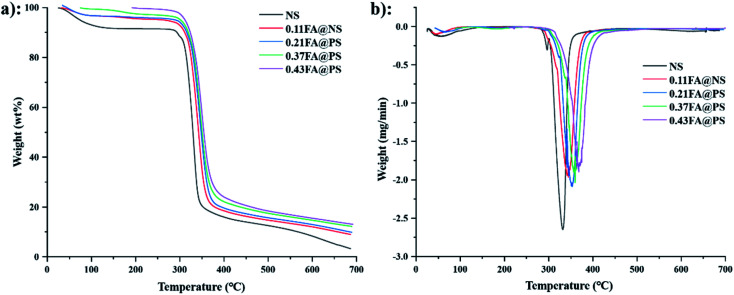
TGA (a) and DTG (b) curves of NS and starch ferulates with different DS. The result indicated that the thermal stability of starch ferulates was increased after esterification.

Thermogravimetric analysis (TGA) and derivative thermogravimetric (DTG) were used to determine the weight loss of the material on heating. NS (Fig. 6a) exhibited a two-stage loss of mass between 50 and 150 °C, which corresponds to an approximate 10% loss of absorbed moisture.^36^ The initial decomposition temperature was 285 °C, and the weight loss reached 50% at 328 °C. The final decomposition temperatures were 331, 343, 352, 358 and 368 °C for NS and the starch ferulates of increasing DS, respectively (Fig. 6b), indicating that esterification with FA increases the thermal stability of starch. The initial weight loss results from the dehydration reaction of intramolecular or intermolecular hydroxyl groups in starch,^37^ and the number of free hydroxyl groups in starch ferulate decreases with increased DS, thereby increasing its thermal stability.

### 3.7. Evaluation of antioxidant capacity

#### 3.7.1 DPPH free radical-scavenging capacity

**Fig. 7:**
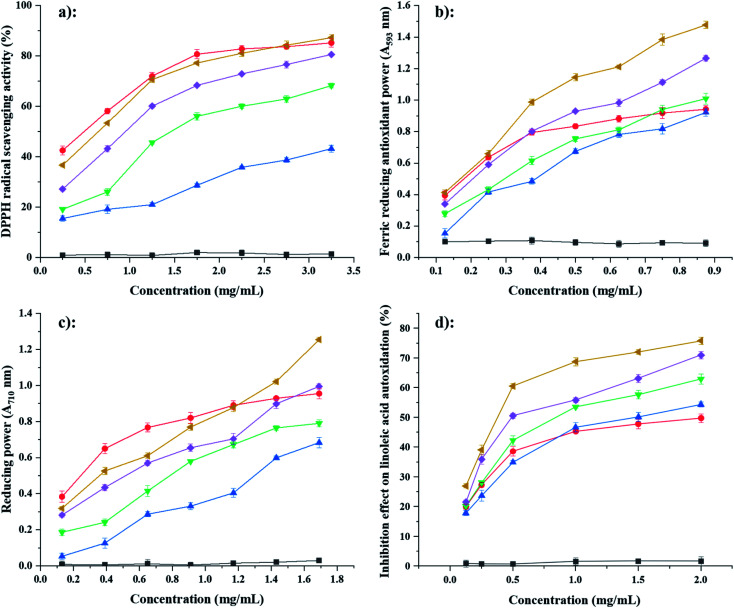
Scavenging activities on 2,2-diphenyl-1-picrylhydrazyl (DPPH) radical (a), ferric reducing antioxidant power (b), reducing power (c) and linoleic acid autoxidation inhibition effect (d) of NS (black, ■), FA (red ●), 0.11FA@NS (blue ▲), 0.21FA@PS (green ▼), 0.37FA@PS (purple ◆) and 0.43FA@PS (yellow ◀). Data are presented as means ± standard deviation of triplicates.

The DPPH assay has been widely used for testing the radical scavenging capacity of plant extracts and antioxidant compounds. The scavenging capacities of FA@PS were markedly higher than that of FA@NS and the scavenging capacity increased with increased DS and concentration (Fig. 7a).^12^ At a concentration of 3.25 mg mL^−1^, the scavenging capacity of 0.43FA@PS (86%) was much higher than those of 0.11FA@NS, 0.21FA@PS and 0.37FA@PS (42%, 68% and 80%, respectively). Among the samples, NS showed the lowest DPPH radical-scavenging ability. After esterification of FA, starch ferulates revealed obviously increased scavenging activity for DPPH radicals but weaker activity than those of free FA.

#### 3.7.2 FRAP assay

The FRAP assay compares the capacity of samples to reduce TPTZ–Fe^3+^ complexes to TPTZ–Fe^2+^ complexes. The results varied similarly to those from the DPPH assay (Fig. 7b). After esterification of FA, starch ferulates revealed greater antioxidant power of ferric reduce. However, the antioxidant power of free FA was weaker activity than those of 0.37FA@PS and 0.43FA@PS when concentration of FA higher 0.4 mg mL^−1^.

#### 3.7.3 Reducing power test

The reducing power of a compound is a significant index of its antioxidant capacity. The results varied similarly to those from the DPPH and FRAP assays, except that the variation was more linear (Fig. 7c).

#### 3.7.4 β-Carotene–linoleic acid assay

The β-carotene–linoleic acid assay is a method for evaluating capacity to inhibit lipid peroxidation. Peroxidation of linoleic acid produces free radicals (lipid hydroperoxides, conjugated diolefins, and volatile by-products), which attack the highly unsaturated β-carotene molecule, disrupting its structure, so its characteristic orange color disappears. The presence of antioxidants inhibits the destruction of the β-carotene conjugated system and the disappearance of the orange color.^20^ The variation in scavenging capacity with concentration was considerably different from the other assays (Fig. 7d), with a very rapid initial increase, followed by a much slower increase above 0.5 mg mL^−1^. The most notable result was that the starch ferulates had a much higher antioxidant capacity than an equal weight (and much higher molar concentration) of FA. This may be explained by formation of chemical bonds, between FA molecules (*via* the phenolic hydroxyl) in the starch ferulates, which would extend the π-bonding conjugated system of FA, making starch ferulates attracting linoleic acid radicals by electrostatic affinity and quenching them, thereby giving starch ferulates a much higher antioxidant capacity than free FA.^38,39^

## 4. Conclusions

**Fig. 8:**
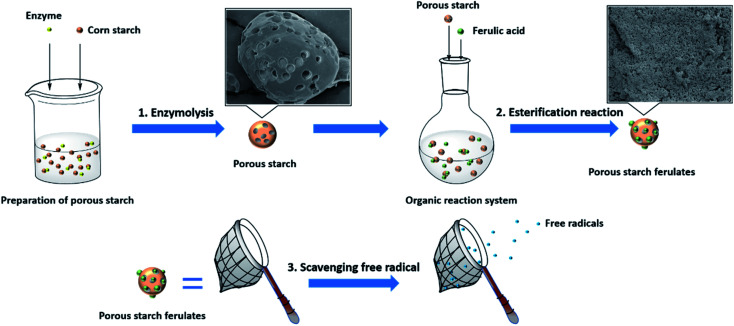
Schematic representation of synthesis and antioxidant capacity of porous starch ferulates.

Ferulic acid modified porous starch esters with a range of DS were successfully synthesized by esterification reaction between hydroxyl groups from PS and carboxyl groups from FA. The schematic diagram for the synthesis and antioxidant capacity of porous starch ferulates is presented in Fig. 8. NMR and FT-IR were utilized to confirm the formation of ester linkages. SEM analysis showed that FA modified PS esters had a different morphology with much more porous structure, which explained the former's much higher DS when esterified with FA. XRD and thermal analysis suggested that starch ferulates was a crystalline material, but with a different crystal structure from the V-type pattern. *In vitro* antioxidant assays showed that starch ferulates had excellent antioxidant capacity. In particular, PS ferulates had a much higher antioxidant capacity than free FA in the β-carotene–linoleic acid assay. PS ferulates have great potential as an antioxidant dietary fiber ingredient in foods, which could protect foods from oxidation and confer health benefits to the consumer. They also have potential as model systems to aid understanding of the interactions of phenolics with other food ingredients. Further work is needed to investigate the toxicology, *in vivo* antioxidant capacity, digestion and metabolism of starch ferulates.

## Conflicts of interest

The authors declare no competing financial interest.

## Supplementary Material
